# An Adjusted Frequency-Domain Algorithm for Arc Array Bistatic SAR Data with One-Moving Transmitter

**DOI:** 10.3390/s22134725

**Published:** 2022-06-23

**Authors:** Pingping Huang, Lingxia Hao, Weixian Tan, Wei Xu, Yaolong Qi

**Affiliations:** 1 College of Information Engineering, Inner Mongolia University of Technology, Hohhot 010051, China; 20191800070@imut.edu.cn (L.H.); wxtan@imut.edu.cn (W.T.); xuwei1983@imut.edu.cn (W.X.); qiyaolong@imut.edu.cn (Y.Q.); 2 Inner Mongolia Key Laboratory of Radar Technology and Application, Hohhot 010051, China

**Keywords:** synthetic aperture radar, arc array antenna, keystone transform, frequency-domain, the spatial resolution

## Abstract

Arc array synthetic aperture radar (AA-SAR), which can observe the scene in all directions, breaks through the single view of traditional SAR. However, the concealment of AA-SAR is poor. To mitigate this, arc array bistatic SAR (AA-BiSAR) with the moving transmitter is proposed, it has the advantages of good concealment and can expand the imaging scene, and improve the flexibility of the system. The imaging geometry including the signal model is established, and a range frequency-domain algorithm based on keystone transform (KT) is proposed in this paper. In the first step, the slant range equation is approximated by Taylor series expansion to compensate for the residual phase caused by the transmitter motion. In the second step, the range cell migration between the range and azimuth is eliminated through the KT method in the range frequency-domain. In the third step, using the data after range cell migration correction in step 2, an azimuth pulse compression is performed to obtain the focused image. In addition, the spatial resolution of the AA-BiSAR system is analyzed in detail. Finally, three simulation results verify the effectiveness of the proposed algorithm and the change in the spatial resolution.

## 1. Introduction

As one of the most distinctive inventions in the 20th century, the helicopter has greatly expanded the application range of aircraft, it can achieve vertical taking-off, landing and hovering at a low altitude. However, the low-altitude environment is complex and changeable, which will affect the ability of the helicopter to observe the surrounding environment directly [1]. In the low-altitude complex environment, whether the helicopter and other airborne craft can perceive the surrounding targets in real-time and detect threats accurately has become a prerequisite for safe flight. The perception equipment for the helicopter is very important for the safe flight, cruise, take-off and landing of the helicopter. Therefore, it is of great significance to study the sensing equipment with real-time sensing and accurate detection of threats.

Arc array synthetic aperture radar (AA-SAR) [2,3,4,5] is a special imaging system proposed in recent years, which can achieve range high resolution by transmitting bandwidth signals, and realizes wide azimuth observation by arc array antenna. Compared with linear array SAR (LA-SAR) [6,7], AA-SAR can quickly image and sense the scene with a wide view angle [4], and can realize the dynamic monitoring and rapid imaging of the surrounding environment. Therefore, AA-SAR imaging system can improve the safe flight of helicopters and has important development potential in military reconnaissance, disaster relief and other fields.

Arc array bistatic SAR (AA-BiSAR) [8] is a new type of variant bistatic SAR system, with the transmitter and receiver deployed on different platforms. Compared with AA-SAR, AA-BiSAR can observe the target from different angles and can obtain front-view or back-view area imaging. Furthermore, the receiver can be designed as a passive system, which can reduce the costs and improve the concealment of the system at the same time. Different configurations of AA-BiSAR can be applied to further enhance the imaging abilities of the system, such as AA-BiSAR with one-moving transmitter. AA-BiSAR with one-moving transmitter can be applied to improve the ability of the helicopter to perceive and detect the surrounding targets, since the transmitter can move, multiple low-cost receivers can share the same transmitter to reduce the system cost. AA-BiSAR with one-moving transmitter has the characteristics of wide observation, good concealment, and especially when the transmitter is moving, it can be used as a mobile tower to assist helicopter flight at the designated point, which improves the flexibility of the system and expands the working area. According to the characteristics, AA-BiSAR with one-moving transmitter can be applied in the helicopter’s auxiliary landing and emergency rescue. When the helicopter is flying, the AA-BiSAR system can ensure the safety of the helicopter with wide observation and high resolution. 

However, due to the special configuration of AA-BiSAR with one-moving transmitter, the raw signal is more challenging to process than the conventional bistatic SAR (BiSAR) [9,10,11] system. On the one hand, different from the traditional invariant BiSAR, the doppler frequency of AA-BiSAR with one-moving transmitter varies not only with the range of target, but also with its azimuth position, which makes the subsequent range cell migration correction (RCMC) [12] more complicated. On the other hand, since the AA-BiSAR system samples at equal angular intervals in the azimuth direction, there will be a trigonometric function under the double root of its slant range equation, so it is difficult to use the principle of stationary phase (POSP) to derive the two-dimensional frequency spectrum directly. In recent years, more and more interest has been devoted to the imaging algorithm of BiSAR with different configurations. For example, an improved nonlinear frequency modulation imaging algorithm is proposed focusing on one-stationary BiSAR, which is combined with motion error compensation to solve the spatial variability of the target point [13]. In addition, the method of series inversion is used to derive the two-dimensional spectrum of the target in bistatic forward-looking SAR [14]. In this paper, regarding AA-BiSAR with one-moving transmitter, a range frequency-domain algorithm was adjusted to the imaging process. First, the residual phase caused by the moving transmitter is corrected in range frequency-domain. Based on this, keystone transform (KT) [15,16,17,18] is used to eliminate the coupling between the range and azimuth, since the imaging capability is greatly affected by the coupling between the range and azimuth. Finally, the focused image is obtained through matched filtering.

The rest of the paper is organized as follows. The working mode, including the imaging geometry and the basic principle of AA-BiSAR with one-moving transmitter are introduced in Section 2. The signal model is analyzed based on the imaging geometry, and the imaging algorithm based on KT is presented in Section 3. In Section 4, the spatial resolution of AA-BiSAR is derived and evaluated in detail. In Section 5, the simulation experiments are carried out to verify the effectiveness of the proposed algorithm. Finally, a conclusion is given in Section 6.

## 2. Arc Array Bistatic SAR System 

AA-SAR as a special form of SAR has the advantage of wide azimuth observation. However, since the transmitter and receiver are deployed on the same platform, the concealment of the system is poor. On the contrary, one-stationary bistatic SAR (OS-BiSAR) has the advantage of good concealment, and it does not have the ability of full azimuth observation. In order to obtain a wide azimuth observation, meanwhile, and improve the concealment of the system, AA-BiSAR with one-moving transmitter is proposed in this paper. AA-BiSAR with one-moving transmitter both has advantages of AA-SAR and OS-BiSAR, which can be applied to the field of helicopter assisted landing and emergency rescue.

**Figure 1:**
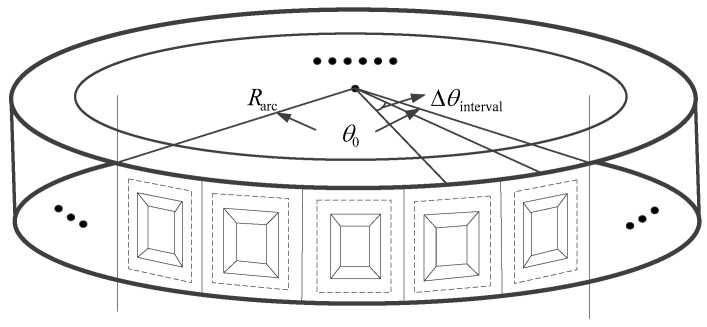
The structure of arc array antenna.

Shown in Figure 1, is the three-dimensional structure diagram of the arc array antenna. A series of antenna array elements are uniformly arranged along the arc direction. The antenna elements are staggered and distributed evenly at equal intervals, and the direction is always from the center of the circle toward the outside of the circle. It can be seen from the structure diagram, $\Delta \theta_{\mathrm{interval}}$ is the angular interval between adjacent equivalent sampling points, $\theta_{0}$ denotes the synthetic aperture angle of the arc array antenna. Let $R_{\mathrm{arc}}$ denotes the radius of the arc array antenna, the synthetic aperture angle and the arc array radius will affect the resolution of the system.

**Figure 2:**
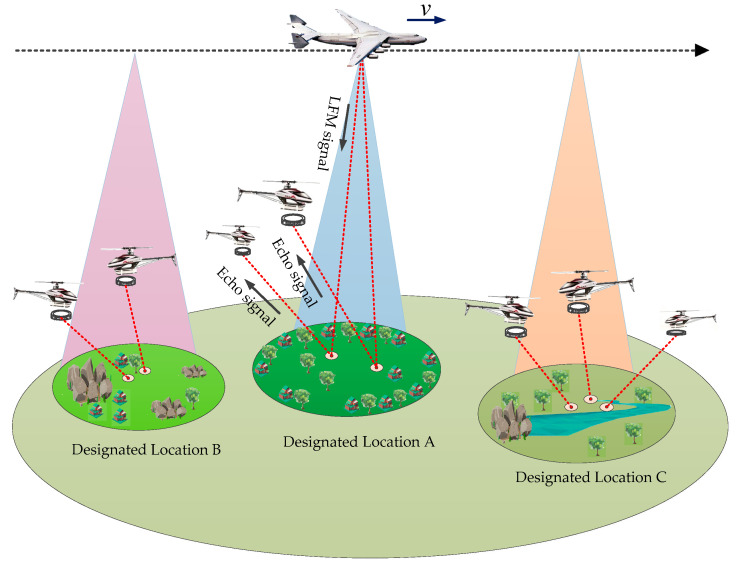
The work mode of AA-BiSAR with one-moving transmitter.

Figure 2 is the work mode of AA-BiSAR with one-moving transmitter. In a large-scale imaging scene, the helicopters are equipped with arc array antenna, and the transmitter platform moves like a mobile tower, waiting to help the helicopters. In order to obtain accurate ground information, the helicopter first sends a request signal to the transmitter, after receiving the request signal, the transmitter moves to the designated location and transmits a high-power chirp signal to the target area. Then, the arc array antennas are turned on in turn to receive the reflected signal of the target, and finally, the ground target information is obtained through imaging processing. In this process, high resolution in azimuth is achieved by synthetic arc aperture, and high resolution in range is achieved by using the transmitted chirp signal. After completing the mission of location A, the transmitter moves to another area to continue assisting other helicopters, which not only ensures the safety of helicopters but also reduces the cost of the system.

**Figure 3:**
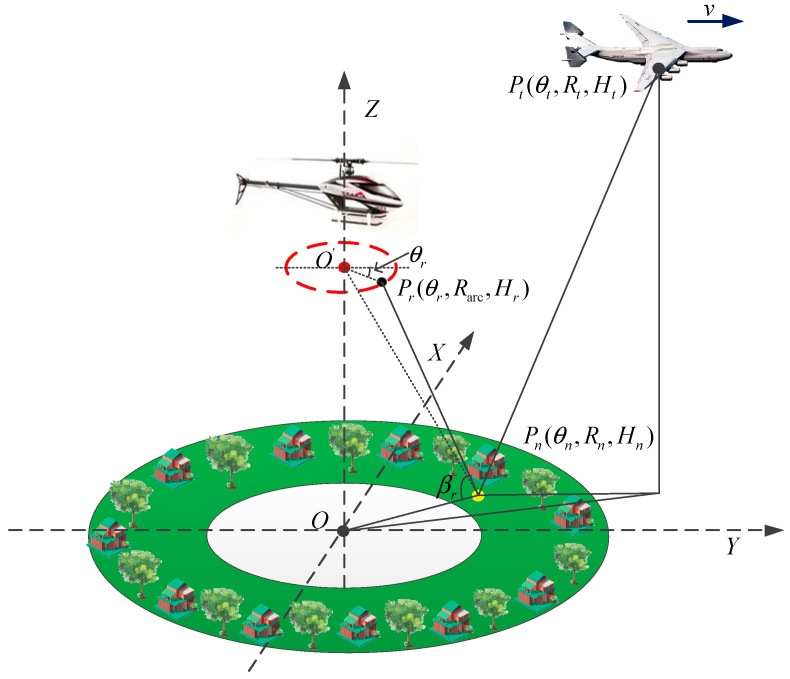
The imaging geometry of AA-BiSAR with one-moving transmitter.

The general imaging geometry of AA-BiSAR with one-moving transmitter is shown in Figure 3. The transmitter moves with a constant velocity v along the Y axis, and $t_{a}$ denotes the azimuth time variable. When $t_{a}=0$, the initial position of the transmitter is $P_{t}(\theta_{t},R_{t},H_{t})$, where $\theta_{t}$ is the azimuth angle of the transmitter, $R_{t}$ stands for the ground distance from the transmitter to the center of the scene, and $H_{t}$ is the height of the transmitter platform. The equivalent sampling point coordinates of the receiver arc array antenna is $P_{r}(\theta_{r},R_{\mathrm{arc}},H_{r})$, where $\theta_{r}$ is the azimuth angle of the equivalent sampling point, $R_{\mathrm{arc}}$ is the antenna radius, and $H_{r}$ represents the height of the receiver platform. Let the coordinates of any point target in the scene be $P_{n}(\theta_{n},R_{n},H_{n})$, where $\theta_{n}$ stands for the azimuth angle of the point target, $R_{n}$ denotes the distance from the point target to the center of the scene, and $H_{n}$ is the height of the point target. Let ρ be the distance from the center of arc array antenna to the point target $P_{n}$, $\beta_{r}$ represents the angle between the ground and $O^{’}P_{n}$.

## 3. Imaging Processing of AA-BiSAR with One-Moving Transmitter 

### 3.1. Signal Model

Without loss of generality, assuming that the receiver switches arc array unit at an angular velocity of $\varpi_{a}$, and $t_{a}$ is the azimuth time variable. $R_{\mathrm{tr}}(t_{a})$ denotes the instantaneous slant range of AA-BiSAR system, it can be described as:

(1) $$\left\{ \begin{array}{l} R_{\mathrm{tr}}(t_{a})=R_{T}+R_{R}\\ R_{T}=\sqrt{{\left(R_{n}\cos\theta_{n}-R_{t}\cos\theta_{t}-vt_{a}\right)}^{2}+{\left(R_{n}\sin\theta_{n}-R_{t}\sin\theta_{t}\right)}^{2}+(H_{n}-H_{t}{)}^{2}}\\ R_{R}=\sqrt{{\left(R_{n}\cos\theta_{n}-R_{r}\cos\theta_{r}\right)}^{2}+{\left(R_{n}\sin\theta_{n}-R_{r}\sin\theta_{r}\right)}^{2}+(H_{n}-H_{r}{)}^{2}}\\ \theta_{r}=\Delta \theta_{\mathrm{interval}}\times n,\;n=1,2,3,\ldots ,N_{r} \end{array}\right.$$

The above Equation (1) is the precise expression of the bistatic slant range. $R_{T}$ and $R_{R}$ are the instantaneous slant ranges from the point target to the transmitter and to the equivalent sampling point, respectively. $N_{r}$ is the total number of the equivalent sampling points in the azimuth direction, and the angular velocity $\varpi_{a}$ is denoted as follows:

(2) $$\varpi_{a}=\frac{\theta_{r}}{t_{a}}$$

Since the instantaneous bistatic slant range contains cosine terms and velocity-related terms, it is difficult to derive an accurate two-dimensional spectrum directly using the POSP, so it is necessary to do some approximates process to resolve the equation. Firstly, expand $R_{T}$ according to the Taylor series, and ignore the higher-order phases above the third order. For ease of expression, let:

(3) $$R_{0}=\sqrt{R_{t}^{2}+R_{n}^{2}+H_{t}^{2}-2R_{t}R_{n}}$$

After being resolved, $R_{T}$ can be expressed as:

(4) $$\begin{array}{ll} R_{T} & \approx \sqrt{R_{0}{}^{2}+2R_{t}R_{n}\left[1-\cos(\theta_{n}-\theta_{t})\right]}-\left(\frac{R_{n}\cos\theta_{n}-R_{t}\cos\theta_{t}}{R_{0}}\right)vt_{a}\\ & +\left[\frac{1}{2R_{0}}-\frac{{\left(R_{n}\cos\theta_{n}-R_{t}\cos\theta_{t}\right)}^{2}}{2R_{0}{}^{3}}\right]{\left(vt_{a}\right)}^{2}+\left(\frac{R_{n}\cos\theta_{n}-R_{t}\cos\theta_{t}}{2R_{0}{}^{3}}\right){\left(vt_{a}\right)}^{3}\\ & -\frac{1}{8R_{0}{}^{3}}{\left(vt_{a}\right)}^{4} \end{array}$$

For the point target $P_{n}$, the distance from $P_{n}$ to the center of arc array $O^{\prime }$ can be written as:

(5) $$\rho =\sqrt{H_{r}{}^{2}+R_{n}{}^{2}}$$

since the arc array radius $R_{r}$«ρ in the practical applications, $R_{R}$ can be resolved as:

(6) $$R_{R}\approx \rho -R_{r}\cos\beta_{r}\cos(\theta_{r}-\theta_{n})$$

After being approximated and resolved, the instantaneous bistatic slant range can be rewritten as:

(7) $$\begin{array}{ll} R_{\mathrm{tr}}(t_{a}) & \approx \rho -R_{r}\cos\beta_{r}\cos(\theta_{r}-\theta_{n})+\sqrt{R_{0}{}^{2}+2R_{t}R_{n}\left[1-\cos(\theta_{n}-\theta_{t})\right]}\\ & -\left(\frac{R_{n}\cos\theta_{n}-R_{t}\cos\theta_{t}}{R_{0}}\right)vt_{a}+\left[\frac{1}{2R_{0}}-\frac{{\left(R_{n}\cos\theta_{n}-R_{t}\cos\theta_{t}\right)}^{2}}{2R_{0}{}^{3}}\right]{\left(vt_{a}\right)}^{2}\\ & +\left(\frac{R_{n}\cos\theta_{n}-R_{t}\cos\theta_{t}}{2R_{0}{}^{3}}\right){\left(vt_{a}\right)}^{3}-\frac{1}{8R_{0}{}^{3}}{\left(vt_{a}\right)}^{4} \end{array}$$

According to Equation (7), the approximated slant range is composed of two parts, where the front part is independent of the velocity of the transmitter, and the latter part is related to the velocity of the transmitter. In order to facilitate the subsequent calculation, let the approximated slant range be expressed as follows:

(8) $$\left\{ \begin{array}{ll} R_{\mathrm{tr}}(t_{a})= & R\left(\theta_{r}\right)+R(t_{a})\\ R\left(\theta_{r}\right)= & \rho -R_{r}\cos\beta_{r}\cos(\theta_{r}-\theta_{n})+\sqrt{R_{0}{}^{2}+2R_{t}R_{n}(1-\cos(\theta_{n}-\theta_{t}))}\\ R(t_{a})= & -\left(\frac{R_{n}\cos\theta_{n}-R_{t}\cos\theta_{t}}{R_{0}}\right)vt_{a}+\left[\frac{1}{2R_{0}}-\frac{{\left(R_{n}\cos\theta_{n}-R_{t}\cos\theta_{t}\right)}^{2}}{2R_{0}{}^{3}}\right]{\left(vt_{a}\right)}^{2}\\ & +\left(\frac{R_{n}\cos\theta_{n}-R_{t}\cos\theta_{t}}{2R_{0}{}^{3}}\right){\left(vt_{a}\right)}^{3}-\frac{1}{8R_{0}{}^{3}}{\left(vt_{a}\right)}^{4} \end{array}\right.$$

In this article, the transmitted chirp signal can be expressed as follows:

(9) $$s_{\mathrm{tr}}(t_{r})=\omega_{r}\left(t_{r}\right)\exp\left(j2\pi f_{c}t_{r}+j\pi K_{r}t_{r}{}^{2}\right)$$

where $\omega_{r}\left(t_{r}\right)$ stands for the rectangular envelope during the time $T_{r}$, $K_{r}$ is the frequency modulation rate, and $f_{c}$ is referred to as the carrier frequency. $t_{r}$ is the range time variable.

After demodulating to baseband, the reflected echo signal from the target is received by an equivalent sampling point, which is given by:

(10) $$\begin{array}{ll} s(t_{r},t_{a})= & A_{0}\omega_{a}\left(\frac{\theta_{r}-\theta_{n}}{\theta_{a}}\right)\omega_{r}\left(t_{r}-R_{tr}(t_{a})/c\right)\\ & \cdot \exp\left[-j\frac{2\pi f_{c}}{c}R_{\mathrm{tr}}(t_{a})\right]\cdot \exp\left\{j\pi K_{r}{\left[t_{r}-\frac{R_{\mathrm{tr}}(t_{a})}{c}\right]}^{2}\right\} \end{array}$$

The above equation is the echo signal of AA-BiSAR with one-moving transmitter, where $\omega_{a}\left(\cdot \right)$ and $\omega_{r}\left(\cdot \right)$ are the azimuth time envelope and range time envelope, respectively. $A_{0}$ is the scattering coefficient of the point target in the scene, $\theta_{a}$ is the array beam width, and *c* is the speed of light.

### 3.2. Residual Phase Compensation

In this section, the residual phase compensation will be deduced to solve part of the range cell migration. Due to the special configuration of AA-BiSAR with one-moving transmitter, the residual phase introduced by the moving of the transmitter will cause severe range cell migration. Therefore, it is necessary to introduce a phase compensation function to correct the residual phase in the range frequency-azimuth time domain. The range frequency-azimuth time domain expression of the echo signal is given by:

(11) $$\begin{array}{ll} S_{s}(f_{r},t_{a})= & A_{0}\omega_{a}\left(\frac{\theta_{r}-\theta_{n}}{\theta_{a}}\right)W_{r}\left(f_{r}\right)\\ & \cdot \exp\left[-j2\pi \frac{\left(f_{r}+f_{c}\right)}{c}\cdot R_{\mathrm{tr}}(t_{a})\right]\cdot \exp\left(-j\pi \frac{f_{r}{}^{2}}{K_{r}}\right) \end{array}$$

where $W_{r}\left(f_{r}\right)$ is the envelope of the signal in the range frequency-domain, and $f_{r}$ is the range frequency variable. Substituting Equation (8) into Equation (11):

(12) $$\begin{array}{ll} S_{s}(f_{r},t_{a})= & A_{0}\omega_{a}\left(\frac{\theta_{r}-\theta_{n}}{\theta_{a}}\right)W_{r}\left(f_{r}\right)\\ & \cdot \exp\left\{-j2\pi \frac{\left(f_{r}+f_{c}\right)}{c}\cdot \left[R\left(\theta_{r}\right)+R(t_{a})\right]\right\}\cdot \exp\left(-j\pi \frac{f_{r}{}^{2}}{K_{r}}\right) \end{array}$$

In order to compensate for the residual phase caused by the moving platform, the compensation function is constructed as follows:

(13) $$H_{c}(f_{r},t_{a})=\exp\left[j2\pi \frac{\left(f_{r}+f_{c}\right)}{c}\cdot R(t_{a})\right]$$

Multiplying Equations (12) and (13), obtained the result as follows:

(14) $$\begin{array}{ll} S_{s1}(f_{r},t_{a})= & A_{0}\omega_{a}\left(\frac{\theta_{r}-\theta_{n}}{\theta_{a}}\right)W_{r}\left(f_{r}\right)\\ & \cdot \exp\left[-j2\pi \frac{\left(f_{r}+f_{c}\right)}{c}\cdot R\left(\theta_{r}\right)\right]\cdot \exp\left(-j\pi \frac{f_{r}{}^{2}}{K_{r}}\right) \end{array}$$

According to Equation (14), it can be seen that after the residual phase is compensated, the raw signal of the target is no longer related to the velocity of the transmitter. Afterward, the range pulse compression is performed, and the matched filter function is constructed by the matched filtering method as follows:

(15) $$H_{r}(f_{r})=\exp\left(j\pi \frac{f_{r}{}^{2}}{K_{r}}\right)$$

After the range pulse compression, the echo signal is compressed as:

(16) $$\begin{array}{ll} S_{sf\_c}(f_{r},t_{a}) & =S_{s1}(f_{r},t_{a})\cdot H_{r}(f_{r})\\ & =A_{0}\omega_{a}\left(\frac{\theta_{r}-\theta_{n}}{\theta_{a}}\right)W_{r}\left(f_{r}\right)\cdot \exp\left[-j2\pi \frac{\left(f_{r}+f_{c}\right)}{c}\cdot R\left(\theta_{r}\right)\right] \end{array}$$

### 3.3. Correction of Range Cell Migration 

According to the analysis in Section 2, after the residual phase is corrected, the slant range is given by:

(17) $$R\left(\theta_{r}\right)=\rho -R_{r}\cos\beta_{r}\cos(\theta_{r}-\theta_{n})+\sqrt{R_{0}{}^{2}+2R_{t}R_{n}(1-\cos(\theta_{n}-\theta_{t}))}$$

To facilitate expression, let $d_{t0}$ defined as follows:

(18) $$d_{t0}=\sqrt{R_{0}{}^{2}+2R_{t}R_{n}(1-\cos(\theta_{n}-\theta_{t}))}$$

Substituting Equation (17) into Equation (16) to resolve the equation, the result is as follows:

(19) $$\begin{array}{ll} S_{sf\_c}(f_{r},\theta_{r})= & A_{0}\omega_{a}\left(\frac{\theta_{r}-\theta_{n}}{\theta_{a}}\right)W_{r}\left(f_{r}\right)\cdot \exp\left[-j2\pi \frac{\left(f_{r}+f_{c}\right)}{c}\cdot (d_{t0}+\rho )\right]\\ & \cdot \exp\left[j2\pi \frac{f_{c}}{c}R_{r}\cos\beta_{r}\cos(\theta_{r}-\theta_{n})\right]\\ & \cdot \exp\left[j2\pi \frac{f_{r}}{c}R_{r}\cos\beta_{r}\cos(\theta_{r}-\theta_{n})\right] \end{array}$$

It can be seen from Equation (19) that the range frequency variable $f_{r}$ and the azimuth angle variable $\theta_{r}$ are coupled in the last term, which will cause severe range cell migration, and lead to the range envelope movement. In this paper, the KT method is proposed to correct the range cell migration by redefining a virtual azimuth variable φ, and it is satisfied in the following relationship:

(20) $$\cos(\varphi -\theta_{n})=\frac{f_{c}+f_{r}}{f_{c}}\cos(\theta_{r}-\theta_{n})$$

After KT processing, the echo signal of the range frequency-azimuth time domain is expressed as:

(21) $$\begin{array}{ll} S_{sf\_k}(f_{r},\varphi )= & A_{0}\omega_{a}\left(\frac{\theta_{r}-\theta_{n}}{\theta_{a}}\right)W_{r}\left(f_{r}\right)\\ & \cdot \exp\left[-j2\pi \frac{f_{c}}{c}(d_{t0}+\rho -R_{r}\cos\beta_{r}\cos(\varphi -\theta_{n})\right]\\ & \cdot \exp\left[-j2\pi \frac{f_{r}}{c}(d_{t0}+\rho )\right] \end{array}$$

Then, perform range inverse fast Fourier transform on Equation (21), and obtain the range time-azimuth time domain expression of echo signal as follows:

(22) $$\begin{array}{ll} s_{r\_k}(t_{r},\varphi ) & =\mathrm{IFFT}\left[S_{sf\_k}(f_{r},\varphi )\right]\;\\ & =A_{0}\omega_{a}\left(\frac{\varphi -\theta_{n}}{\theta_{a}}\right)\phi_{r}\left(t_{r}-\frac{d_{t0}+\rho }{c}\right)\\ & \cdot \exp\left[-j2\pi \frac{f_{c}}{c}(d_{t0}+\rho )\right]\cdot \exp\left[j2\pi \frac{f_{c}}{c}R_{r}\cos\beta_{r}\cos(\varphi -\theta_{n})\right] \end{array}$$

where IFFT [·] stands for the inverse fast Fourier transform operation, $\phi_{r}\left[t_{r}-(d_{t0}+\rho /c)\right]$ is the inverse Fourier transform of $W_{r}\left(f_{r}\right)$. It can be seen from Equation (22), after KT processing, that the coupling between the range and azimuth is eliminated, and the echo envelopes of different range cells are corrected to the same range cell. 

### 3.4. Azimuth Pulse Compression

It is necessary to perform azimuth inverse Fourier transform on the echo signal of Equation (22), and the matched filtering of azimuth needs to be performed in the range doppler domain. The azimuth matched filtering can be realized by the fast convolution, mainly to copy the pulse perform Discrete Fourier Transform (DFT) after zero padding. Then, taking the complex conjugate of the result, the convolution kernel can be expressed as:

(23) $$h_{a}(\varphi )=\exp\left[j2\pi \frac{f_{c}}{c}R_{r}\cos\beta_{r}\cos(\varphi -\theta_{n})\right]$$

So, the matched filtering function of azimuth can be calculated as:

(24) $$H_{\mathrm{az}}(f_{\varphi })={\left\{\mathrm{FFT}\left[h_{a}(\varphi )\right]\right\}}^{*}$$

where ${\left[\;\right]}^{*}$ stands for the complex conjugate operation. After azimuth matched filtering, the echo signal is finally focused as:

(25) $$\begin{array}{ll} s_{r\_\mathrm{ka}}(t_{r},\varphi ) & =\mathrm{IFFT}\{(\mathrm{FFT}(s_{r\_k}(t_{r},\varphi )))\times H_{\mathrm{az}}(f_{\varphi })\}\\ & =A_{0}\phi_{a}(\varphi -\theta_{n})\phi_{r}\left(t_{r}-\frac{d_{t0}+\rho }{c}\right)\cdot \exp\left[-j2\pi \frac{f_{c}}{c}(d_{t0}+\rho )\right] \end{array}$$

where $\phi_{a}(\varphi -\theta_{n})$ denotes the envelope of azimuth direction. 

**Figure 4:**
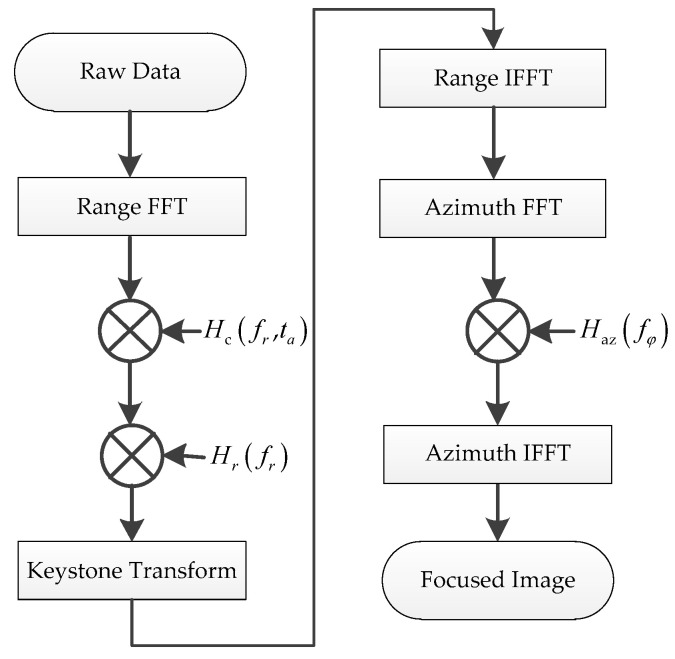
The imaging flowchart of AA-BiSAR with one-moving transmitter.

The complete imaging flowchart of AA-BiSAR with one-moving transmitter is shown in Figure 4. After the residual phase caused by the moving transmitter is compensated in the range frequency-azimuth time domain, the echo signal is no longer related to the velocity of the transmitter. Then, using keystone transform, the coupling between the range frequency and azimuth angle is successfully eliminated, and the focused images are output after azimuth matched filtering.

## 4. Resolution Analysis 

The spatial resolution is one of the important indicators to evaluate the effectiveness of radar imaging algorithms, which reflects the ability of radar to distinguish targets in different locations. The azimuth resolution of AA-BiSAR is mainly determined by the arc synthetic aperture and the relative position between the receiver and target. The range resolution is mainly achieved by the transmission bandwidth signal. In this paper, the spatial resolution of AA-BiSAR with the moving transmitter is analyzed in detail, and the spatial resolution of the moving receiver is studied.

### 4.1. AA-BiSAR with Moving Transmitter

#### 4.1.1. Azimuth Resolution 

The azimuth resolution of AA-BiSAR is also called the azimuth angular resolution, the unit generally is an angle, which can be derived from the echo signal of the point target. According to the analysis in the above section, the raw data of AA-BiSAR with one-moving transmitter system is given by:

(26) $$\begin{array}{ll} s(t_{r},t_{a})= & A_{0}\omega_{a}\left(\frac{\theta_{r}-\theta_{n}}{\theta_{a}}\right)\omega_{r}\left(t_{r}-R_{\mathrm{tr}}(t_{a})/c\right)\\ & \cdot \exp\left[-j2\pi \frac{f_{c}}{c}R_{\mathrm{tr}}(t_{a})\right]\cdot \exp\left\{j\pi K_{r}{\left[t_{r}-\frac{R_{\mathrm{tr}}(t_{a})}{c}\right]}^{2}\right\} \end{array}$$

since the arc array radius $R_{r}$«ρ in the practical applications, the distance from the target to the receiver can be resolved as $R_{R}=\rho -R_{r}\cos\beta_{r}\cos(\theta_{r}-\theta_{n})$, so the bistatic slant range expression can be shown as:

(27) $$\begin{array}{ll} R_{\mathrm{tr}}(t_{a})= & R_{R}+R_{T}\\ & \approx \rho -R_{r}\cos\beta_{r}\cos(\theta_{r}-\theta_{n})\\ & +\sqrt{{\left(R_{n}\cos\theta_{n}-R_{t}\cos\theta_{t}-vt_{a}\right)}^{2}+{\left(R_{n}\sin\theta_{n}-R_{t}\sin\theta_{t}\right)}^{2}+(H_{n}-H_{t}{)}^{2}} \end{array}$$

and the instantaneous azimuth phase is given by:

(28) $$\psi_{a}=-\frac{2\pi f_{c}}{c}R_{\mathrm{tr}}(t_{a})$$

the instantaneous azimuth frequency can be expressed as:

(29) $$f_{\theta }=\frac{d(\psi_{a})}{d\theta_{r}}=-\frac{2\pi R_{r}\cos\beta_{r}\sin(\theta_{r}-\theta_{n})}{\lambda }+\frac{2\pi v(R_{n}\cos\theta_{n}-R_{t}\cos\theta_{t}-vt_{a})}{\lambda \varpi_{a}R_{T}}$$

where $\varpi_{a}$ is the angular velocity. It can be seen from Equation (29) that the instantaneous azimuth frequency is composed of two parts, one part is caused by the synthetic aperture, and the other part is caused by the movement of the transmitter. Due to the value range of $\left(\theta_{r}-\theta_{n}\right)$ is less than or equal to half of the azimuth beam width of the receiver, which is $\theta_{a}/2$, and $\theta_{a}$ is generally less than 180°. In general, the monotonicity of the instantaneous azimuth frequency is guaranteed, so the instantaneous azimuth frequency changes monotonically. Therefore, the azimuth angular resolution of AA-BiSAR with one-moving transmitter imaging system can be calculated as:

(30) $$\rho_{\theta }=\frac{2\pi }{\max(f_{\theta })-\min(f_{\theta })}$$

Afterward, in order to compare the influences of different transmitter velocities on the azimuth resolution, four groups of comparative experiments are carried out. The detailed simulation parameters of AA-BiSAR with one-moving transmitter system are given in Table 1. The transmitter velocities are set as 0 m/s, 50 m/s, 300 m/s, and in order to amplify the influence of transmitter movement on the azimuth resolution, this article adds a set of experiments with a transmitter velocity of 1000 m/s. The initial position of the transmitter $\left(X_{t},Y_{t},H_{t}\right)$ is set as (0 m, 100 m, 1000 m), and the height of the transmitter and receiver platform are set as 1000 m and 200 m since the height of the aircraft should be much larger than the helicopters for better observe the whole scene. The arc array radius of the receiver platform is set as 0.6 m, which is much smaller than the scene size.

**Figure 5:**
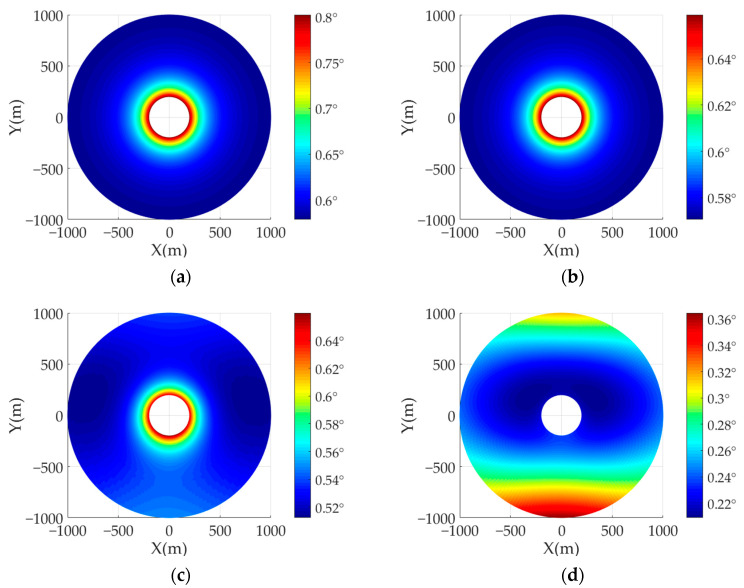
The azimuth angular resolution of AA-BiSAR with one-moving transmitter: (**a**) the transmitter velocity is set as 0 m/s; (**b**) the transmitter velocity is set as 50 m/s; (**c**) the transmitter velocity is set as 300 m/s; (**d**) the transmitter velocity is set as 1000 m/s.

Figure 5 shows the specific simulation results of four comparative experiments, where Figure 5a is the azimuth resolution when the transmitter is stationary at a certain point. It can be observed that the azimuth resolution of the target is only related to the radius of the arc array antenna, the synthetic aperture angle and the height of the receiver platform. In short, when the transmitter is stationary, the azimuth resolution has nothing to do with the target azimuth angle, and for those targets with the same radius, the azimuth resolution remains unchanged. Figure 5b–d is the azimuth resolution of the target, which varies with the transmitter speed. Since, when the transmitter is moving, the azimuth frequency of the point target consists of two parts: the synthetic aperture formed by the curved array antenna, and the synthetic aperture formed by the moving transmitter. As a result, the azimuth angular resolution of targets is not only related to the radius of the arc array antenna and synthetic aperture angle, but also to the instantaneous position of the transmitter. Furthermore, the greater the velocity of the transmitter, the more obvious the change in azimuth angular resolution, and as the squint angle between the target point and the transmitter becomes larger, the azimuth angular resolutions of targets become worse. In the simulation experiment, since the azimuth time variable is set within the time of an arc synthetic aperture, when the transmitter speed is small, the targets azimuth resolution changes little, as the transmitter speed increases, the azimuth resolution is getting better within a certain range.

#### 4.1.2. Ground-Range Resolution

The range resolution reflects the ability of radar to distinguish two close targets, which is mainly determined by the bandwidth of the transmitted signal. AA-BiSAR with one-moving transmitter, the transmitter and receiver are separated. Different from traditional monostatic SAR, the instantaneous slant range of AA-BiSAR comes from two platforms, the range of a certain platform can not be expressed as the bistatic range alone. Therefore, in this article, the ground-range resolution of AA-BiSAR with one-moving transmitter is analyzed via the gradient method. 

Assuming that the initial coordinates of the transmitter, receiver, and the point target are: $P_{t}=(X_{t},Y_{t},Z_{t})$, $P_{r}=(X_{r},Y_{r},Z_{r})$, $P_{n}=(X_{n},Y_{n},Z_{n})$, converted to the cylindrical coordinates as follows:

(31) $$\left\{ \begin{array}{l} X_{t}=R_{t}\sin\theta_{t},\;Y_{t}=R_{t}\cos\theta_{t},\;Z_{t}=H_{t}\\ X_{r}=R_{r}\sin\theta_{r},\;Y_{r}=R_{r}\cos\theta_{r},\;Z_{r}=H_{r}\\ X_{n}=R_{n}\sin\theta_{n},\;Y_{n}=R_{n}\cos\theta_{n},\;Z_{n}=H_{n} \end{array}\right.$$

The bistatic slant range of point target at azimuth time $t_{a}$ is expressed as:

(32) $$\begin{array}{ll} R_{b}= & \sqrt{{\left(X_{n}-X_{r}\right)}^{2}+{\left(Y_{n}-Y_{r}\right)}^{2}+{\left(Z_{n}-Z_{r}\right)}^{2}}\\ & +\sqrt{{\left(X_{n}-X_{t}\right)}^{2}+{\left(Y_{n}-Y_{t}-vt_{a}\right)}^{2}+{\left(Z_{n}-Z_{t}\right)}^{2}} \end{array}$$

The gradient of any point target $\left(X_{n},Y_{n}\right)$ on the isometric line is given by:

(33) $$\nabla R_{b}=\left(\frac{X_{n}-X_{t}}{R_{T}}+\frac{X_{n}-X_{r}}{R_{R}}\right)i+\left(\frac{Y_{n}-Y_{t}-vt_{a}}{R_{T}}+\frac{Y_{n}-Y_{r}}{R_{R}}\right)j_{y}$$

where $R_{T}$ is the instantaneous slant range from the transmitter to the target position, and $R_{R}$ is the instantaneous slant range from the target position to the receiver. Let $\xi_{t}$ and $\xi_{r}$ denote the squint angles of the transmitter and receiver. Define $\phi_{t}$ and $\phi_{r}$ as the side-looking angles of the transmitter and receiver, they are expressed as follows:

(34) $$\xi_{t}=\arcsin\left[\frac{Y_{n}-Y_{t}-vt_{a}}{\sqrt{{\left(X_{n}-X_{t}\right)}^{2}+{\left(Y_{n}-Y_{t}-vt_{a}\right)}^{2}+{\left(Z_{n}-Z_{t}\right)}^{2}}}\right]$$

(35) $$\xi_{r}=\arcsin\left[\frac{Y_{n}-Y_{r}}{\sqrt{{\left(X_{n}-X_{r}\right)}^{2}+{\left(Y_{n}-Y_{r}\right)}^{2}+{\left(Z_{n}-Z_{r}\right)}^{2}}}\right]$$

(36) $$\phi_{t}=\arcsin\left[\frac{X_{n}-X_{t}}{\sqrt{{\left(X_{n}-X_{t}\right)}^{2}+{\left(Z_{n}-Z_{t}\right)}^{2}}}\right]$$

(37) $$\phi_{r}=\arcsin\left[\frac{X_{n}-X_{r}}{\sqrt{{\left(X_{n}-X_{r}\right)}^{2}+{\left(Z_{n}-Z_{r}\right)}^{2}}}\right]$$

Let the gradient be expressed by the squint angle and the side-looking angle, the gradient equation and gradient size of the point target can be obtained as follows:

(38) $$\nabla R_{b}=(\sin\phi_{t}\cos\xi_{t}+\sin\phi_{r}\cos\xi_{r}){\vec{i}}_{x}+(\sin\xi_{t}+\sin\xi_{r}){\vec{i}}_{y}$$

(39) $$\left|\nabla R_{b}\right|=\sqrt{{\left(\sin\phi_{t}\cos\xi_{t}+\sin\phi_{r}\cos\xi_{r}\right)}^{2}+{\left(\sin\xi_{t}+\sin\xi_{r}\right)}^{2}}$$

According to Equations (38) and (39), it can be concluded that the ground-range resolution of AA-BiSAR with one-moving transmitter system is expressed as follow:

(40) $$\rho_{r}=\frac{c/B_{r}}{\left|\nabla R\right|}=\frac{c/B_{r}}{\sqrt{{\left(\sin\phi_{t}\cos\xi_{t}+\sin\phi_{r}\cos\xi_{r}\right)}^{2}+{\left(\sin\xi_{t}+\sin\xi_{r}\right)}^{2}}}$$

where $B_{r}$ is the signal bandwidth, *c* is the speed of light. It can be seen from Equation (40) that the ground-range resolution of the system is not only related to the position of the target and receiver, but also related to the position of the transmitter, which is different when the transmitter is fixed.

**Figure 6:**
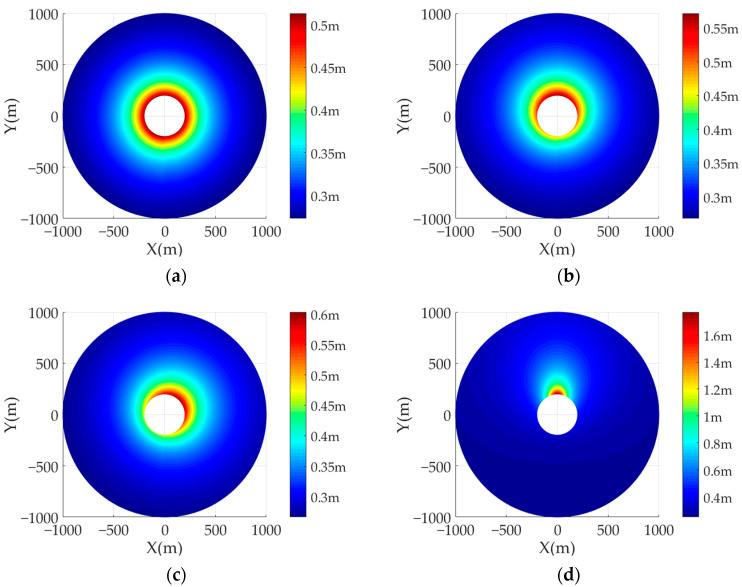
The ground-range resolution of AA-BiSAR with one-moving transmitter: (**a**) the transmitter position is set as (0 m, 0 m, 1000 m); (**b**) the transmitter position is set as (0 m, 100 m, 1000 m); (**c**) the transmitter position is set as (100 m, 100 m, 1000 m); (**d**) the transmitter position is set as (0 m, 700 m, 1000 m).

In the following, in order to analyze the influence of the transmitter position on the ground-range resolution of the system, four sets of comparative experiments are designed. The detailed simulation parameters of AA-BiSAR with one-moving transmitter system are shown in Table 1. The transmitter velocity is set as 50 m/s, and the initial positions of the transmitter are set as (0 m, 0 m, 1000 m), (0 m, 100 m, 1000 m), (100 m, 100 m, 1000 m) and (700 m, 0 m, 1000 m), respectively. Figure 6a–d shows the ground-range resolution results of different targets with different transmitter positions. According to the simulation results, the closer the transmitter to the point targe, the worse the ground-range resolution, and when the transmitter is far away from the target, the ground-range resolution is mainly determined by the distance between the point target and the transmitter position.

### 4.2. The Spatial Resolution of AA-BiSAR with Moving Receiver 

Assuming that the transmitter is fixed directly above the scene, and the receiver moves with speed *v* along the Y axis, the coordinates of the transmitter, the receiver and the point target are as follows:

(41) $$\left\{ \begin{array}{l} P_{t}=\left(0,0,H_{t}\right)\\ P_{r}=\left(\theta_{r},R_{arc},H_{r}\right)\\ P_{n}=\left(\theta_{n},R_{n},H_{n}\right) \end{array}\right.$$

After *t_a_* time, the instantaneous bistatic slant range is obtained as follows:

(42) $$\left\{ \begin{array}{l} R_{pt}=\sqrt{{\left(R_{n}\cos\theta_{n}\right)}^{2}+{\left(R_{n}\sin\theta_{n}\right)}^{2}+{\left(H_{n}-H_{t}\right)}^{2}}\\ R_{pr}=\sqrt{{\left(R_{n}\cos\theta_{n}-R_{arc}\cos\theta_{r}-vt_{a}\right)}^{2}+{\left(R_{n}\sin\theta_{n}-R_{arc}\sin\theta_{r}\right)}^{2}+{\left(H_{n}-H_{r}\right)}^{2}} \end{array}\right.$$

where $R_{pt}$ is the distance from the transmitter to the target, $R_{pr}$ is the distance from the target to the receiver.

As in the above section to analyze AA-BiSAR with the moving transmitter, this section uses the same method to analyze the spatial resolution when the receiver is moving. The unit of azimuth resolution is angle, and the azimuth instantaneous phase can be calculated by the following formula:

(43) $$\psi_{a}=\frac{2\pi f_{c}}{c}R_{\mathrm{tr}}(t_{a})$$

where $R_{\mathrm{tr}}(t_{a})$ is the bistatic slant range, and the specific expression is as follows:

(44) $$\begin{array}{ll} R_{\mathrm{tr}}\left(t_{a}\right) & =\sqrt{{\left(R_{n}\cos\theta_{n}-R_{arc}\cos\theta_{r}-vt_{a}\right)}^{2}+{\left(R_{n}\sin\theta_{n}-R_{arc}\sin\theta_{r}\right)}^{2}+{\left(H_{n}-H_{r}\right)}^{2}}\\ & +\sqrt{{\left(R_{n}\cos\theta_{n}\right)}^{2}+{\left(R_{n}\sin\theta_{n}\right)}^{2}+{\left(H_{n}-H_{t}\right)}^{2}} \end{array}$$

The azimuth instantaneous frequency can be obtained by approximate derivation of Equation (43) as follows:

(45) $$f_{\theta }=\frac{d(\psi_{a})}{d\theta_{r}}=\frac{2\pi R_{r}\cos\beta_{r}\sin(\theta_{r}-\theta_{n})}{\lambda }+\frac{2\pi v(vt_{a}-R_{n}\cos\theta_{n})}{\lambda \omega_{a}\rho }$$

where ρ is the distance from the point target to the center point of the arc array, the specific value is $\rho =\sqrt{H_{r}{}^{2}+R_{n}{}^{2}}$, and $\cos\beta_{r}=\frac{R_{n}}{\rho }$. The range of the azimuth angle variable $\left(\theta_{r}-\theta_{n}\right)$ is 0°~90°, which ensures the monotonicity of the azimuth instantaneous frequency. Therefore, the azimuth resolution of AA-BiSAR with the moving receiver is as follows:

(46) $$\rho_{\theta }=\frac{2\pi }{\max(f_{\theta })-\min(f_{\theta })}\approx \frac{\lambda }{2R_{r}\cos\beta_{r}\sin\left(\theta_{a}/2\right)}+\frac{\lambda \omega_{a}{}^{2}\rho }{v^{2}\theta_{a}}$$

Convert the coordinates of the transmitter, the receiver and the point target to polar coordinates as follows:

(47) $$\left\{ \begin{array}{l} X_{t}=0,\;Y_{t}=0,\;Z_{t}=H_{t}\\ X_{r}=R_{r}\sin\theta_{r},\;Y_{r}=R_{r}\cos\theta_{r},\;Z_{r}=H_{r}\\ X_{n}=R_{n}\sin\theta_{n},\;Y_{n}=R_{n}\cos\theta_{n},\;Z_{n}=H_{n} \end{array}\right.$$

The bistatic slant range at time *t_a_* can be expressed as:

(48) $$\begin{array}{ll} R_{b}= & \sqrt{{\left(X_{n}-X_{r}\right)}^{2}+{\left(Y_{n}-Y_{r}-vt_{a}\right)}^{2}+{\left(Z_{n}-Z_{r}\right)}^{2}}\\ & +\sqrt{X_{n}{}^{2}+Y_{n}{}^{2}+{\left(Z_{n}-Z_{t}\right)}^{2}} \end{array}$$

According to Equation (48), the gradient expression of any target on the equidistant line can be obtained as follows:

(49) $$\nabla R_{b}=\left(\frac{X_{n}}{R_{pt}}+\frac{X_{n}-X_{r}}{R_{pr}}\right)i+\left(\frac{Y_{n}}{R_{pt}}+\frac{Y_{n}-Y_{r}-vt_{a}}{R_{pr}}\right)j_{y}$$

where the values of $R_{pt}$ and $R_{pr}$ are as follows:

(50) $$\left\{ \begin{array}{l} R_{pt}=\sqrt{X_{n}{}^{2}+Y_{n}{}^{2}+{\left(Z_{n}-Z_{t}\right)}^{2}}\\ R_{pr}=\sqrt{{\left(X_{n}-X_{r}\right)}^{2}+{\left(Y_{n}-Y_{r}-vt_{a}\right)}^{2}+{\left(Z_{n}-Z_{r}\right)}^{2}} \end{array}\right.$$

The gradient size is:

(51) $$\left|\nabla R_{b}\right|=\sqrt{{\left(\frac{X_{n}}{R_{pt}}+\frac{X_{n}-X_{r}}{R_{pr}}\right)}^{2}+{\left(\frac{Y_{n}}{R_{pt}}+\frac{Y_{n}-Y_{r}-vt_{a}}{R_{pr}}\right)}^{2}}$$

Therefore, it can be deduced that the ground-range resolution along the gradient direction is:

(52) $$\rho_{r}=\frac{c/B_{r}}{\left|\nabla R_{b}\right|}=\frac{c/B_{r}}{\sqrt{{\left(\frac{X_{n}}{R_{pt}}+\frac{X_{n}-X_{r}}{R_{pr}}\right)}^{2}+{\left(\frac{Y_{n}}{R_{pt}}+\frac{Y_{n}-Y_{r}-vt_{a}}{R_{pr}}\right)}^{2}}}$$

**Figure 7:**
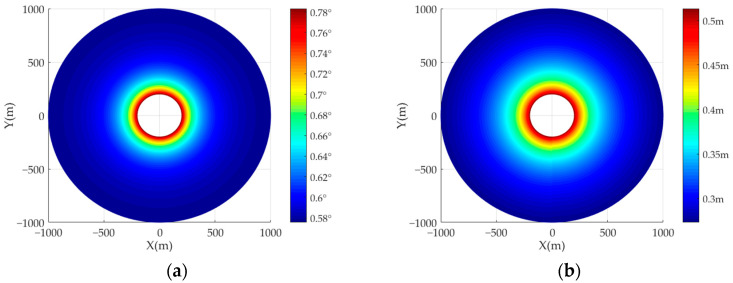
The spatial resolution of AA-BiSAR with moving receiver: (**a**) the azimuth resolution; (**b**) the ground-range resolution.

The simulation is carried out on the spatial resolution of AA-BiSAR with the moving receiver. The position of the receiver is set as (0 m, 0 m, 200 m), and the transmitter position is (0 m, 0 m, 1000 m), and the speed of the receiver is 50 m/s. Figure 7a is the simulation result of the azimuth resolution of different point targets. It can be seen that the azimuth resolution of the point targets of the same radius is basically the same. Figure 7b shows the ground-range resolution of different point targets in the imaging scene. It can be seen that the farther the point target is from the transmitter, the better the ground-range resolution.

## 5. Simulation Results

**Figure 8:**
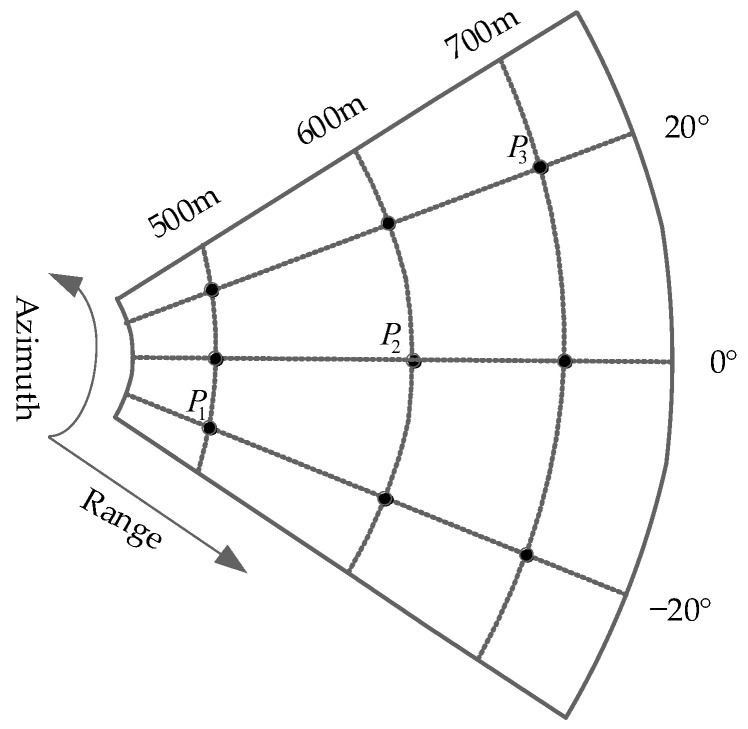
The positions of the simulated targets.

According to the analysis in Section 4, it can be concluded that the velocity and position of the transmitter will affect the spatial resolution of the system. In this section, in order to verify the effectiveness of the algorithm proposed in this paper and observe the impact of transmitter position and velocity changes on the spatial resolution, three sets of simulation experiments are carried out. The detailed simulation parameters are listed in Table 1, and the initial position and velocity of the transmitter are set as in Table 2. In the simulation, the imaging algorithm proposed in this paper is used to simulate the echo of point targets, a total of 9 point targets are simulated in the image scene, Figure 8 shows the distributing positions of the simulated targets. By comparing the imaging performance of different transmitter positions and velocities, the imaging performance of AA-BiSAR with one-moving transmitter can be further understood. 

**Figure 9:**
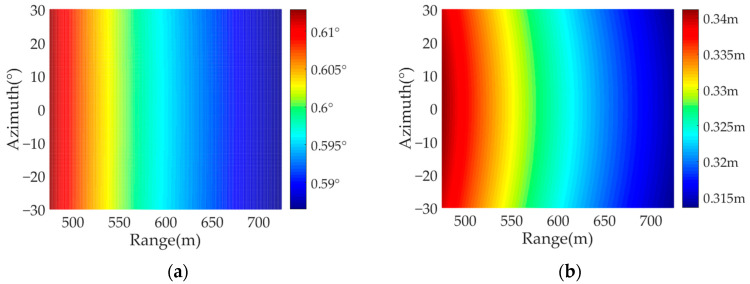

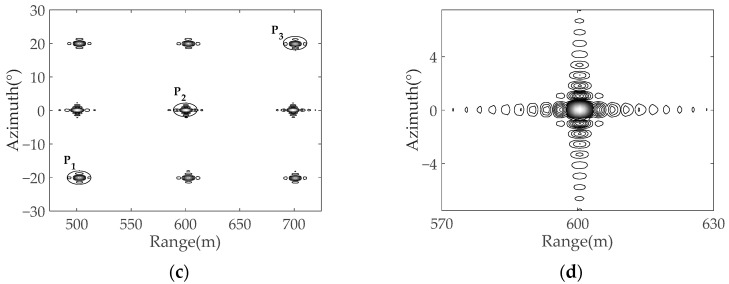
The simulation results of Experiment 1: (**a**) the azimuth resolution; (**b**) the ground-range resolution; (**c**) the imaging results of the simulated targets; (**d**) the contour of P_2_.

Experiment 1: In this experiment, the transmitter initial position is set as (0 m, 100 m, 1000 m), and the velocity of the transmitter is set as 50 m/s. Figure 9 shows the simulation results of Experiment 1, where Figure 9a is the azimuth resolution of the simulated target scene, Figure 9b is the ground-range resolution of the scene, and Figure 9c is the imaging results of the simulated targets. In order to further verify the effectiveness of the proposed algorithm, the point targets P1, P2, and P3 are analyzed in detail. Their position coordinates are set as: (500 m, −20°), (600 m, 0°), (700 m, 20°), respectively. Figure 9d shows the contour of the simulated target P2, it can be observed that the target has good focusing behavior both in the range and azimuth direction.

**Figure 10:**
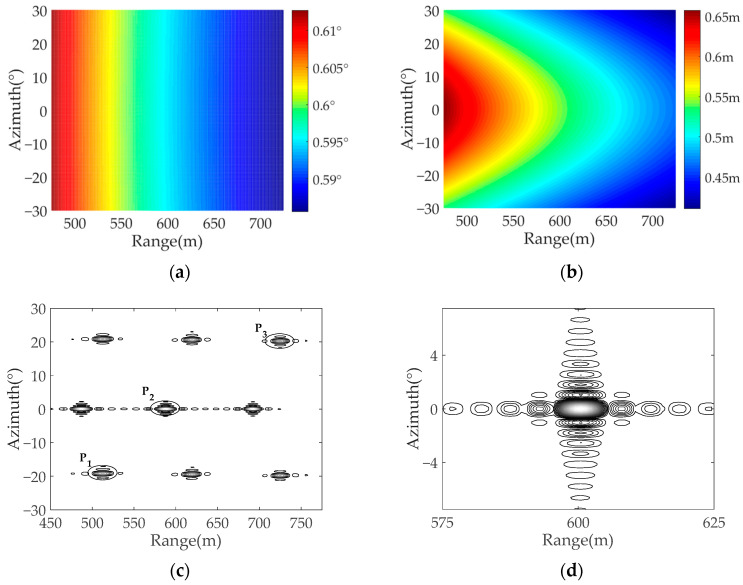
The simulation results of Experiment 2: (**a**) the azimuth resolution; (**b**) the ground-range resolution; (**c**) the imaging result of the simulated targets; (**d**) the contour of P_2_.

Experiment 2: In order to observe the influence of transmitter position change on the spatial resolution, the transmitter initial position is designed as (0 m, 700 m, 1000 m) in this experiment, and the velocity of the transmitter is the same as Experiment 1. The corresponding simulation results of Experiment 2 are shown in Figure 10, it can be observed from Figure 10c that the imaging focusing ability of the simulated targets is significantly reduced, which confirmed that the closer the transmitter is to the target, the worse the ground-range resolution. Figure 10d is the contour of the simulated target, it can be found that the ground-range resolution in Experiment 2 is worse than in Experiment 1, since the position of the transmitter in Experiment 2 is closer to the targets. In addition, due to the distance between the transmitter and receiver becoming larger, there will be imaging distortion in Experiment 2.

**Figure 11:**
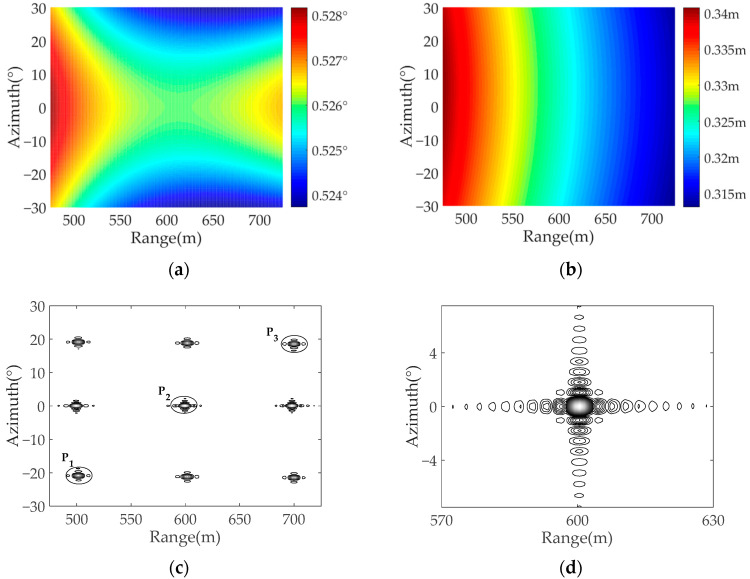
The simulation results of Experiment 3: (**a**) the azimuth resolution; (**b**) the ground-range resolution; (**c**) the imaging results of the simulated targets; (**d**) the contour of P_2_.

Experiment 3: In this experiment, the transmitter velocity is changed to 300 m/s, and the position of the transmitter is set as (0 m, 100 m, 1000 m). Figure 11 shows the simulation results, since the azimuth time variable is set within an arc synthetic aperture time in the simulation, the azimuth resolution of the target changes little. Figure 11d is the contour of the simulated target in Experiment 3, it can be seen that the target focusing ability is better than in the other two group experiments. 

Table 3 provides specific imaging performance indicators, where PSLR and ISLR represent peak sidelobe ratio and integrated sidelobe ratio, respectively. In Experiment 1, the theoretical values of the ground-range resolution of P_1_, P_2_, and P_3_ are 0.354 m, 0.331 m and 0.312 m, respectively, and the theoretical values of the azimuth angular resolution are 0.608°, 0.596° and 0.588°. According to the result data in Table 3, it is concluded that the performance imaging result of the point target is basically consistent with the theoretical analysis value, which verifies the effectiveness of the algorithm proposed in this paper.

In Experiment 2, the theoretical values of the ground-range resolution of P_1_, P_2_, and P_3_ are 0.569 m, 0.543 m and 0.448 m, respectively, and the theoretical values of the azimuth angular resolution are 0.608°, 0.595° and 0.587°. Compared with the data in Experiment 1, it can be concluded that when the transmitter position changes, there is almost no impact on the azimuth resolution of the system, however, it will have a great impact on the ground-range resolution.

In Experiment 3, the theoretical values of the ground-range resolution of P_1_, P_2_, and P_3_ are 0.352 m, 0.330 m, and 0.312 m, respectively, and the theoretical values of the azimuth angular resolution are 0.529°, 0.528°, and 0.527°. Compared with the data in Experiment 1, it can be seen that the velocity of the transmitter has almost no impact on the range-ground resolution, however, it will impact the azimuth resolution of the system, when the transmitter velocity is increased from 50 m/s to 300 m/s, the azimuth resolution has improved.

## 6. Conclusions

In order to expand the imaging scene and improve the flexibility of the system, a new configuration of AA-BiSAR with one-moving transmitter is proposed in this paper. Compared with AA-SAR and one-stationary BiSAR, AA-BiSAR with one-moving transmitter has the characteristics of wide observation, well concealment and flexible structure. It has important research significance in helicopter assisted landing and emergency rescue. The imaging geometry and the echo signal model were established. To obtain the focused image, an imaging algorithm based on the KT method is proposed. In the imaging algorithm, the residual phase caused by the moving transmitter is compensated in the range frequency-domain. Then, the coupling between the range and azimuth is eliminated via the KT method by redefining a new azimuth variable. As a result, the raw data of the system can be well focused. Furthermore, the spatial resolution of the system is also analyzed, the transmitter position affects the ground-range resolution, and the transmitter speed affects the azimuth resolution. The experiment results verify the effectiveness of the proposed algorithm and the variation of the spatial resolution.

## Figures and Tables

**Table 1:** Simulation parameters.

Symbol	Definition	Value
$f_{c}$	Carrier Frequency	50.5 GHz
$B_{r}$	Signal Bandwidth	650 MHz
$T_{r}$	Sweep Time	0.15 ms
$H_{r}$	The Height of Receiver	200 m
$R_{\mathrm{arc}}$	The Radius of Arc Array	0.6 m
$\varpi_{a}$	Velocity of Azimuth Angular	30 rad/s
$\theta_{a}$	Array Beam Width (−3 dB)	60°

**Table 2:** Different parameters of the three group experiments.

Experiment Number	$\mathrm{Transmitter}\;\mathrm{Position}\;(X_{t},Y_{t},H_{t})$	Transmitter Speed Value
1	(0 m, 100 m, 1000 m)	50 m/s
2	(0 m, 700 m, 1000 m)	50 m/s
3	(0 m, 100 m, 1000 m)	300 m/s

**Table 3:** Measured parameters of the selected targets.

Experiment Number	Target	Range				Azimuth			
		Resolutions (m)		PSLR (dB)	ISLR (dB)	Resolutions (°)		PSLR (dB)	ISLR (dB)
		Theoretical	Actual			Theoretical	Actual		
1	P_1_	0.354	0.357	−13.273	−9.754	0.608	0.613	−12.210	−8.618
	P_2_	0.331	0.339	−12.965	−9.393	0.596	0.598	−12.523	−8.706
	P_3_	0.312	0.314	−13.204	−9.678	0.588	0.589	−11.985	−8.473
2	P_1_	0.569	0.571	−13.330	−9.755	0.608	0.610	−11.328	−8.200
	P_2_	0.543	0.544	−13.319	−9.749	0.595	0.600	−12.546	−8.698
	P_3_	0.448	0.449	−13.252	−9.774	0.587	0.592	−12.168	−8.640
3	P_1_	0.352	0.353	−13.326	−9.767	0.529	0.531	−12.046	−8.744
	P_2_	0.330	0.333	−12.916	−9.416	0.528	0.529	−12.522	−8.692
	P_3_	0.312	0.313	−13.189	−9.673	0.527	0.531	−11.114	−8.377

## Data Availability

Not applicable.
